# Chimpanzees and Humans Mimic Pupil-Size of Conspecifics

**DOI:** 10.1371/journal.pone.0104886

**Published:** 2014-08-20

**Authors:** Mariska E. Kret, Masaki Tomonaga, Tetsuro Matsuzawa

**Affiliations:** 1 Primate Research Institute, Kyoto University, Inuyama, Aichi, Japan; 2 Department of Psychology, University of Amsterdam, Amsterdam, the Netherlands; 3 Amsterdam Brain and Cognition (ABC) center, Amsterdam, the Netherlands; University of Tuebingen Medical School, Germany

## Abstract

Group-living typically provides benefits to individual group members but also confers costs. To avoid incredulity and betrayal and allow trust and cooperation, individuals must understand the intentions and emotions of their group members. Humans attend to other's eyes and from gaze and pupil-size cues, infer information about the state of mind of the observed. In humans, pupil-size tends to mimic that of the observed. Here we tested whether pupil-mimicry exists in our closest relative, the chimpanzee (*P. troglodytes*). We conjectured that if pupil-mimicry has adaptive value, e.g. to promote swift communication of inner states and facilitate shared understanding and coordination, pupil-mimicry should emerge within but not across species. Pupillometry data was collected from human and chimpanzee subjects while they observed images of the eyes of both species with dilating/constricting pupils. Both species showed enhanced pupil-mimicry with members of their own species, with effects being strongest in humans and chimpanzee mothers. Pupil-mimicry may be deeply-rooted, but probably gained importance from the point in human evolution where the morphology of our eyes became more prominent. Humans' white sclera surrounding the iris, and the fine muscles around their eyes facilitate non-verbal communication via eye signals.

## Introduction

Many daily social decisions are made through quick evaluations of others. The ability to recognize emotions is one of the most important factors involved in regulating social interactions in primates [1].Through face-to-face interactions, humans and chimpanzees learn to recognize characteristics (such as emotional expressions and group membership) that signal safety, and to cooperate with those that seem trustworthy [2]. According to the theory of emotional contagion, another's state may be perceived *through* synchronization or mimicry [3]. Mimicry is shared among the great apes [4], allows individuals to mirror other's minds [5], [6] and has positive effects on the bond between individuals [7], [8], [9].

Synchronization occurs at different levels, ranging from *body postures* and *facial expressions* [10], [11] to physiological states, such as *heart-beat rythm* [12], [13], *neural activity* [14], or *eye-blinks* [15]. Research has also shown mimicry of *pupil-size* [16], [17], a phenomenon dubbed pupillary contagion that is linked to empathy and involves activity of the amygdala [18], [19], an area densely connected with brain-stem areas that regulate the pupil [20]. This interesting phenomenon has only received little attention in the literature and many questions have remained unanswered about the exact meaning of pupil-size mimicry.

The meaning of pupil-dilation is also diverse and can reflect many inner states including attention, arousal, cognitive effort and interest [21]. Importantly, there is a qualitative difference between the *expression* and the *perception* of pupil-size. Large pupils are perceived positively. In the 17th century, Italian women through the use of atropine-containing eyedrops induced pupil dilation to appear more attractive. Also Japanese manga- and Disney illustrators have since long applied this common knowledge “that large pupils are good” successfully in their cartoons (bambi has large pupils, the big bad woolf pin-point pupils). Despite this anecdotal evidence, the positive impression that large pupils evoke has only received very little scientific attention thus far. We here propose that in the context of a social interaction, pupil-dilation may implicitly signal *social interest*, which would explain why large pupils in an observed induce positive feelings and pupil-mimicry in an observer [16].

The positive effects of mimicry are particularly relevant for members within the same group, as group cohesion and cooperation are important predictors for survival [22]. Previous research has indeed shown that mimicry occurs more often between two members of the same group than between members from different groups [23], [24], [25], even on automatic levels such as heart-beat, which is particularly interesting as it cannot be controlled or regulated [26], [27]. From the literature, it seems that *how* group is defined is not of much influence. A group setting, whether defined by political attitudes, sport affiliation [24], status [28], familiarity [23], ethnicity [25] or species [29], impacts on the *perceived similarity* between observer and observed, which enhances mimicry. The current study is the first in investigating within-species advantages on pupil-mimicry and in investigating an autonomic form of synchronization in two different species.

In humans, the eyes are important in understanding another's intentions [30] and capture more attention than any other area of the face [31]. The typical *white color of our sclera* facilitated non-vocal communication [32] which has recently been demonstrated in a series of experimental studies [33]. Lee et al (2013) suggest that our eye-white served to *link* expresser and observer (see also [34]). Eye-white appeared after the human and chimpanzee lineages split six million years ago and may have served as a *catalyst* for new forms of eye communication to emerge, including pupil-mimicry. Chimpanzees have no visible eye-white but do have bright amber-colored irises in which the pupil is clearly visible. Not only in humans, but across different species, pupil-dilation indicates attention and arousal [35]. For that reason, one could also argue that picking up pupillary cues from a conspecific would be beneficial for all species that live in social groups, that cooperate and are active during daytime.

In order to investigate the *uniqueness* of pupil-mimicry for the human species, we here make a direct comparison with the chimpanzee. If chimpanzees show pupil-mimicry, this would suggest that it existed in our common ancestor and may even be shared by other primates or mammals. Chimpanzees form a perfect comparison group as they are most closely related to us, are known to synchronize on other more explicit levels (laughing, [36]; yawning [28], [29], [37], [38]; tapping with a sound [39] and also mimic familiar others more than unfamiliar others [38]. Moreover, they have similar visual acuity as humans [40], a similar pupil-response [41], they attend to eyes [42], and although less automatically than humans, also follow eye-gaze [43], [44], [45].

In the current study, eighteen human and eight chimpanzee subjects watched four-second video-clips of the eye-region of both species. The pupils in these images either slightly dilated or constricted and with eye-tracking equipment, we measured whether subject's pupils mimicked the pupils they observed. We predicted that chimpanzees would synchronize their pupils with chimpanzees and not with humans. As human eyes are ideally set up for communication, we predicted that humans would show even stronger pupil-mimicry effects than chimpanzees, and specifically with observed human eyes.

## Method

### Participants

Eight chimpanzees (two female and one male juvenile, four female and one male adult) and eighteen humans (seven male) participated in this study. Chimpanzees were 24.63 years old on average (SD = 11.54) and human subjects were on average 25.83 years old (SD = 8.40). At the time of testing, the chimpanzees lived within a social group of fourteen individuals in an enriched environment with a 700-m2 outdoor compound and an attached indoor residence that was illuminated during day-time at the Kyoto University Research Unit in Inuyama, Japan. The outdoor compound was equipped with climbing frames, ropes, small streams, and various species of trees. Access to the outdoor compound was available to them every other day during the day. Daily meals included a wide variety of fresh fruits and vegetables fed throughout the day supplemented with nutritionally balanced biscuits (fed twice daily) and water available ad libitum. The chimpanzees have been familiar with humans since birth and interact with them on a daily basis (especially during feeding and prior to and after the experiments). They have taken part in various cognitive experiments since youth [34], [39], [40]. For the daily experiments, the chimpanzees left the social group voluntarily on the request of experimenters, moved into the experimental booth with the guidance of experimenters, and moved back to the social group after the completion of experiments (approx. 1 hour). The human participants were scientists or students from Kyoto University (fourteen Asian, four Caucasian). Sixteen had experience with the observation of chimpanzees. As the chimpanzees in our sample were very familiar with humans, our aim was to include humans that were also familiar with chimpanzees. Human subjects provided written informed consent prior to the experiment and received full debriefing upon completion of the study. The study with human participants reported here was reviewed and approved by the Human Research Ethics Committee, Primate Research Institute, Kyoto University (H2010-17). The care and use of the chimpanzees adhered to the 3rd edition of the Guide for the Care and Use of Laboratory Primates issued by Primate Research Isntitute, Kyoto University (KUPRI) in 2010, which is compatible with the guidelines issued by the National Institute of Health in the United States of America. The research design was approved by the Animal Welfare and Animal Care Committee of KUPRI and by the Animal Research Committee of Kyoto University (#2011-078). All procedures adhered to the Japanese Act on Welfare and Management of Animals.

### Apparatus

**Figure 1:**
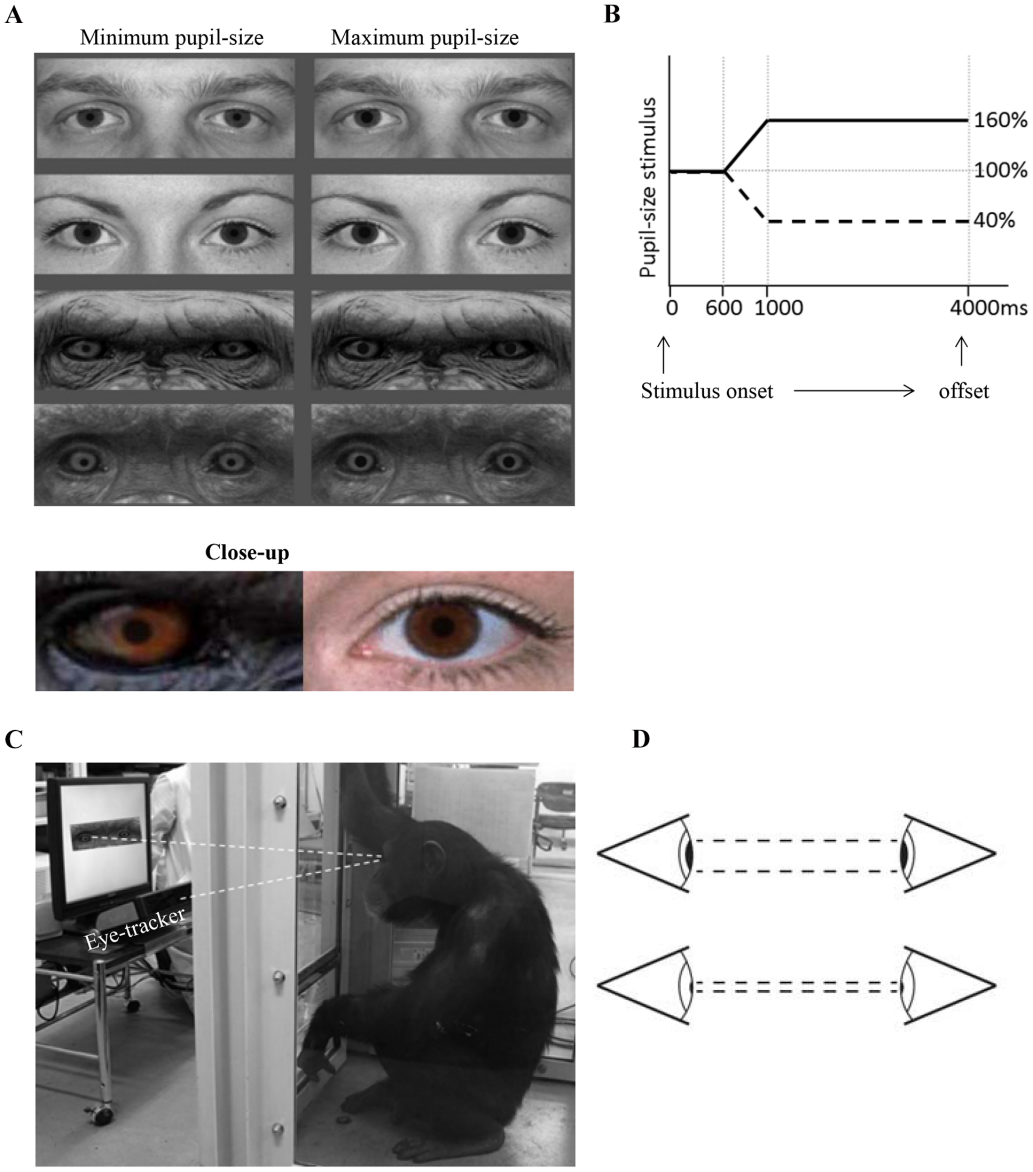
Experimental Set-up. **A/B.** Stimulus' pupils changed dynamically in size after 600 ms of static presentation. The maximum or minimum was reached after 1000 ms. **C.** The testing situation was the same for human and chimpanzee subjects. **D.** Schematic representation of pupil-size mimicry.

We used a binocular eye tracker that allowed for relatively large head movements of participants during tracking (60 Hz; Tobii ×120). The chimpanzees had experience with eye-tracking [43]. A five- and two-point calibration was applied in humans and chimpanzees subjects respectively. A corneal reflection technique was applied. All participants were tested in the same experimental booth, with the experimenter and the participant separated by transparent acrylic panels. The distance between the participants and the movable table on which the eye-tracker and a 17-inch LCD display were mounted was adjusted to the point at which gaze was best recorded (approximately 60 cm). See Figure 1.

### Stimulus Materials

We prepared pictures from four chimpanzees and four humans (two males, two females). The human faces were selected from the McArthur set (www.macbrain.org) and the chimpanzee pictures were shot at the chimpanzee sanctuary in Kumamoto, Japan. The individuals depicted in the stimuli were all unfamiliar to the participants. We first measured the average luminance of the pixels in the irises to make sure that it was the same for the human and chimpanzee pictures so that pupillary changes were equally visible in the human and chimpanzee stimuli (Mean luminance chimpanzee iris  = 201 vs. Mean human iris  = 212). The pictures were then edited in Adobe Photoshop to crop the eye region, remove the pupil and make them life-size. The reason for cropping the pictures was to minimize possible confounding differences in eye-gaze patterns across the two species. In addition, the average luminance of the pixels covering the whole eye region was measured for each stimulus, to be able to include these values in our statistical model. In Adobe After Effects, an artificial pupil was created which started to dilate or contract for 400 ms after 600 ms of static presentation. During the final three seconds of stimulus presentation, the pupil-size was static and smaller (40%) or larger (160%) as compared to the beginning of stimulus presentation. The size and dilation speed were based on measurements in human subjects in extreme lighting conditions (a flashlight in a dark room). We made the pupils in the stimuli start to change after 600 ms of static presentation because we wanted to create the impression in the participant that the change in the pupil-size in the stimulus was *a reaction related to the observed watching him/the participant* (as in a social interaction). For this same reason, we chose dynamic stimuli and not static pictures where pupils were either large or small. The stimuli were presented against a grey background, which had the same luminance as the average of all stimuli. See Figure 1.

### Procedure

After successful calibration, the first trial was presented. A trial started with a fixation cross on which subjects had to focus for 250 ms, followed by a stimulus, i.e., a pair of life-size eyes. Chimpanzees were given two random trials a day, after which they were rewarded with small pieces of fruit. Trials were repeated if subjects got distracted during the presentation of a stimulus [42], [43]. Human subjects completed all trials in one session. The three juvenile chimpanzees in our sample were 11 years old and were never separated from their mothers. As always, also during the current experiment, they were in the same testing booth but did not disturb each other. If the juvenile was involved in our eye-tracking experiment, the mother was performing an unrelated, non-social task on a touch-screen in the same testing booth at a distance of approximately three meter (and vise-versa).

### Experimental Design/Statistical Analyses

The experiment had a 2×2×2 design (subject species (2), looking at their own or the other species (2), with the stimulus pupils either dilating or constricting (2)).

There are multiple ways to analyze pupil-size. While the most common way is to average pupil-size over stimulus presentation time, some researchers prefer to analyze the peak amplitude [46]. Both indices are informative, but effects on the *slope* of the pupil response cannot be detected when pupil-size is averaged over time or when just one time-point (the peak) is selected. Fortunately, there is a more complete *and* precise analysis method which allows us to compare the intercept and the steepness and curvature of the slope of participant's pupil size over time. For that purpose, multi-level models have been suggested as the most appropriate analysis for any type of psychophysiological study, including pupillometry [47]. With this statistical method it is possible to include all sampled data-points in the analysis without the necessity to average over trials, time-points or even the two eyes. That way, all variance in the data is maintained, nested data is accounted for, and with the possibility to include fixed and random factors, the statistical model can be set up in a way so that it most optimally explains the variance in the data.

In order to analyze participant's pupil-size over time, we used a four-level regression model (Linear Mixed Model, implemented in SPSS) [47], [48], [49]. The multi-level structure is defined by the repeated measures, i.e., time (level 1), nested in trials (level 2), nested in eyes (level 3), nested in participants (level 4). Time was included as a repeated factor with a First-Order Autoregressive (AR1) covariance structure to control for auto-correlation with regard to time. As we were specifically interested in the effect of the stimulus pupil size on the pupil response of the subject and not in direct comparisons between human and chimpanzee stimuli or subjects, we group-centered the pupil response in order to be able to better interpret the intercept as a function of pupil mimicry. Group-centering does not affect the model fit nor does it impact on the interactions.

We first included the following fixed predictors in the model: Species Subject, Species Stimulus, Pupil-size Stimulus, Linear Term, Quadratic Term, Cubic Term and all their interactions (i.e., the factors of primary interest), but also Luminance of Stimulus, Trial and Stimulus individual (i.e., factors not of primary interest but which could potentially modulate pupil-size). As a common method, and in order to get the most parsimonious model with the best fit, non-significant effects were removed one by one, starting with the higher-order interactions. Via likelihood ratio tests, we verified whether the removal of a non-significant factor improved the fit of the model or not, or significantly decreased model fit. In the latter case, the factor was kept, otherwise it was excluded. This is the most standard model-selection procedure [47], [48], [49]. After specifying the fixed effects, model building proceeded with statistical tests of the variances of the random effects. The following random predictors were included: Subject individual, Subject individual * Stimulus Individual and Linear, Quadratic and Cubic Terms. Below we explain the procedures for the fixed and random effects separately, with all the considerations and tests that were made. The final factors that endured all these tests were kept in the final model that is shown in Table 1.

**Table 1:** Final model human and chimpanzee subjects.

Type III Tests of Fixed Effects							
Source	Numerator	Denominator df	F	Sig.			
Intercept	1	388.617	3.997	**0.046**			
Lin	1	52.515	40.492	**0.000**			
Pupil-size Stimulus	1	374.441	0.621	0.431			
Species Subject	1	337.706	2.917	0.089			
Own/Other Species	1	368.324	4.346	**0.038**			
Lin * Pupil-size Stimulus	1	353.329	1.102	0.294			
Lin * Species Subject	1	51.723	2.693	0.107			
Lin * Own/Other Species	1	344.961	0.037	0.847			
Pupil-size Stimulus * Species Subject	1	372.082	1.458	0.228			
Pupil-size Stimulus * Own/Other Species	1	371.83	0.000	0.984			
Species Subject * Own/Other Species	1	367.83	2.618	0.107			
Lin * Pupil-size Stimulus * Species Subject	1	348.939	0.464	0.496			
Lin * Pupil-size Stimulus * Own/Other Species	1	335.653	2.558	0.111			
Lin * Species Subject * Own/Other Species	1	349.287	1.335	0.249			
Pupil-size Stimulus * Species Subject * Own/Other Species	1	369.921	5.816	**0.016**			
Lin * Pupil-size Stimulus * Species Subject * Own/Other Species	1	336.842	5.703	**0.017**			
Quad	1	49.423	42.081	**0.000**			
Quad * Pupil-size Stimulus	1	365.48	8.035	**0.005**			
Quad * Species Subject	1	45.267	2.156	0.149			
Quad * Own/Other Species	1	357.35	0.585	0.445			
Quad * Pupil-size Stimulus * Species Subject	1	354.967	4.796	**0.029**			
Quad * Pupil-size Stimulus * Own/Other Species	1	349.82	13.124	**0.000**			
Quad * Species Subject * Own/Other Species	1	357.433	27.084	**0.000**			
Cub	1	35.771	15.955	**0.000**			
Cub * Species Subject	1	35.332	2.868	0.099			
Cub * Own/Other Species	1	358.649	0.236	0.627			
Cub * Species Subject * Own/Other Species	1	356.59	12.727	**0.000**			
Luminance	1	367.921	3.902	**0.049**			

Estimates of Covariance Parameters							
Parameter						95% Conf. Interv.	
		Estimate	Std. Error	Wald Z	Sig.	Lower Bound	Upper Bound
Repeated Measures	AR1 diagonal	0.020	0.000	39.631	**0.000**	0.019	0.021
	AR1 rho	0.970	0.001	1264.654	**0.000**	0.968	0.971
	Var:						
Intercept + Lin + Quad + Cub	Intercept	0.029	0.002	12.406	**0.000**	0.025	0.034
[subject = id * Stimulus Number]	Lin	0.430	0.058	7.35	**0.000**	0.329	0.561
	Quad	0.266	0.034	7.852	**0.000**	0.207	0.342
	Cub	0.104	0.016	6.326	**0.000**	0.076	0.141
Intercept + Lin + Quad + Cub	Intercept	0.011	0.004	3.052	**0.002**	0.006	0.021
[subject = id]	Lin	0.321	0.104	3.083	**0.002**	0.17	0.605
	Quad	0.167	0.055	3.063	**0.002**	0.088	0.317
	Cub	0.057	0.021	2.752	**0.006**	0.028	0.115

#### Fixed factors

In order to capture the nonlinear change of pupil size, we included linear, quadratic and cubic terms for the time factor in the model. All these terms were significant. We included Species of Subject (human vs. chimpanzee), Species Stimulus (own vs. different species), Pupil-size Stimulus (dilating vs. constricting) in the model and as there was a four-way interaction with the Linear Term and two three-way interactions between Species Subject * Own/Other Species * Quadratic Term and Species Subject * Own/Other Species * Cubic Term respectively, all lower-order effects had to be kept in the model too.

We had four different human pictures and four different chimpanzee pictures. To test whether stimulus individual had any unforeseen effect on the higher order interactions, we created dummy codes and included them in the model. As the significance of the higher order interactions were unaffected by stimulus individual, we could omit stimulus individual as a fixed predictor from the model.

#### Random factors

We considered random effects of the overall intercept, overall linear, overall quadratic and overall cubic term and random effects of the intercept, linear, quadratic and cubic terms associated with each stimulus individual. All were significant which means that individual differences were present, i.e., some subjects had a larger pupil response than others or had a steeper increase, a higher peak, or the pattern showed a clearer cubic pattern than was the case for other individual subjects. The variance in the data clustered around these individual patterns and also around each unique stimulus seen by each unique individual. See Table 1 for the final model and the statistics.

#### Predictions

We had four à priori predictions. We predicted that (i) subjects would have a larger pupil-size when observing pupils that dilate vs. constrict in their own vs. the other species; (ii) subject's pupil-sizes would increase faster, i.e., have a steeper slope in the condition where stimulus' pupils dilate vs. constrict, especially when observing their own species; (iii) subject's pupil-response would show a greater bell-shaped curve or peak when observing stimulus' pupils that dilate vs. constrict, especially when they observe their own species. Of importance is the *relative* difference in participant's pupil-size/change when stimulus' pupils dilate vs. constrict and not so much changes as compared to baseline. The presentation of any new stimulus to the eye initiates sudden changes in neural activity in the visual cortex which causes initial pupil constriction. The pupils take +-6 seconds to recover which is why participant's pupil size will dilate in all our stimulus conditions.

As pupil-mimicry can have potential benefits in both species, we expected to find these interactions in both, but predicted stronger effects in humans, given their particularly communicative eyes with the most ideal morphology for detecting eye signals (iv). In statistical terms, we expected to find interactions between (i) Own/Other Species Stimulus × Pupil-size Stimulus; (ii) Own/Other Species Stimulus × Pupil-size Stimulus *×* Linear Term and (iii) Own/Other Species Stimulus × Pupil-size Stimulus × Quadratic Term. We finally predicted that these three interactions would be further qualified by Species Subject (iv). Below we will only report on effects that include the factor Pupil-size Stimulus. *All* effects are listed in Table 1.

## Results

### Pupil-Mimicry

There was no interaction between Own/Other Species Stimulus × Pupil-size Stimulus (i) or between Own/Other Species Stimulus × Pupil-size Stimulus × Linear term (ii) (*p*s >.111). However, these two interactions were both further qualified by Species Subject *F* = 5.703, *p = *.017; *F* = 5.816, *p = *.016. In line with our prediction, there was a strong interaction between Own/Other Species Stimulus × Pupil-size Stimulus × Quadratic Term *F* = 13.124, *p*<.0005 that was not further qualified by Species Subject. See Table 1.

**Figure 2:**
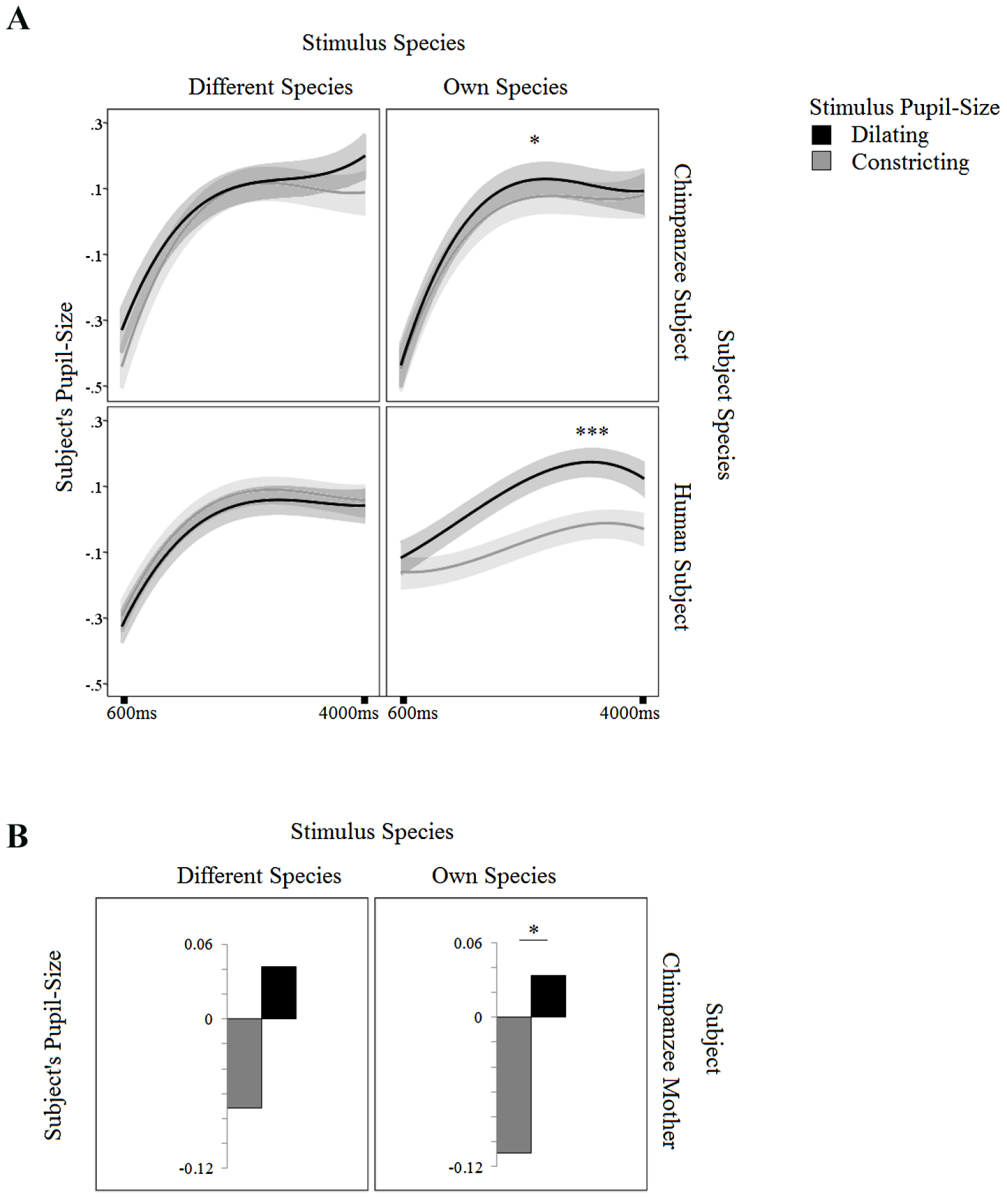
Pupil-Mimicry. **A.** In human subjects, a strong effect of mimicry -specifically with the pupils in human eyes- was observed. The chimpanzee subjects also showed pupil-mimicry with their own group, but only in terms of the quadratic effect and not on the average pupil-size. **B.** The chimpanzee mothers in our sample, as humans, did show an effect on the intercept. Their pupils were larger when they observed chimpanzees with dilating vs. constricting pupils.

We further investigated these effects in separate models within each species. Human subjects showed a significant interaction between Stimulus Pupil-size and Own/Other Species Stimulus *F* = 17.399, *p<*.001, indicating that their pupils synchronized with the pupils as observed in human eyes, but not in chimpanzee eyes. Interestingly, both human and chimpanzee subjects showed an interaction between the Quadratic Term, Stimulus Pupil-size and Own/Other Species Stimulus (humans *F* = 8.231, *p = *.004; chimpanzees *F* = 4.631, *p = *.034. The interaction demonstrates that both species showed a greater peak in their pupil size when observing a member of their own species with pupils that dilated vs. constricted. See Figure 2A and Table 2.

**Table 2:** Final model for humans.

Type III Tests of Fixed Effects							
Source	Numerator	Denom. df	F	Sig.			
Intercept	1	268.941	8.579	**0.004**			
Lin	1	35.650	48.510	**0.000**			
Pupil-size Stimulus	1	253.120	0.897	0.345			
Own/Other Species	1	252.455	4.434	**0.036**			
Lin * Pupil-size Stimulus	1	251.578	0.215	0.643			
Lin * Own/Other Species	1	254.067	6.161	**0.014**			
Pupil-size Stimulus * Own/Other Species	1	253.405	17.399	**0.000**			
Lin * Pupil-size Stimulus * Own/Other Species	1	251.910	3.672	0.056			
Quad	1	33.314	49.516	**0.000**			
Quad * Pupil-size Stimulus	1	252.212	0.154	0.695			
Quad * Own/Other Species	1	252.735	54.352	**0.000**			
Quad * Pupil-size Stimulus * Own/Other Species	1	253.086	8.231	**0.004**			
Cub	1	24.220	11.512	**0.002**			
Cub * Own/Other Species	1	246.465	65.304	**0.000**			
Luminance	1	252.461	8.389	**0.004**			

Estimates of Covariance Parameters							
Parameter						95% Conf. Interv.	
		Estimate	Std. Error	Wald Z	Sig.	Lower Bound	Upper Bound
Repeated Measures	AR1diag gdiag.	0.009	0.000	45.916	**0.000**	0.009	0.010
	AR1rho	0.943	0.001	76.678	**0.000**	0.941	0.946
Int+ Lin+ Quad+Cub	Int	0.033	0.003	1.989	**0.000**	0.027	0.039
[subject = id *Stimulus Nr]	Lin	0.605	0.064	9.412	**0.000**	0.491	0.745
	Quad	0.283	0.032	8.782	**0.000**	0.226	0.354
	Cub	0.089	0.013	6.667	**0.000**	0.067	0.120
Int+ Lin+ Quad+Cub	Int	0.012	0.005	2.553	**0.011**	0.006	0.026
[subject = id]	Lin	0.281	0.111	2.537	**0.011**	0.130	0.609
	Quad	0.173	0.066	2.640	**0.008**	0.082	0.364
	Cub	0.056	0.022	2.569	**0.010**	0.026	0.120

### Differences between Chimpanzee Individuals

Eight unique chimpanzee individuals were tested. Inspection of the individual patterns showed that the three mothers in our sample (Ai, Chloe and Pan), showed the strongest pupil-mimicry, especially when viewing their own species. When running the final chimpanzee model with just these three individuals included, a main effect of Stimulus Pupil-size was observed when viewing chimpanzees with pupils that dilated vs. constricted *F* = 5.455, *p = *.028. A trend towards a late pupil-mimicry effect was found when these three mothers observed human eyes with pupils that dilated vs. constricted *F* = 3.128, *p = *.091. See Figure 2B. See Table 3.

**Table 3:** Final model for chimpanzees.

Type III Tests of Fixed Effects							
Source	Numerator	Denom. df	F	Sig.			
Intercept	1	14.400	0.132	0.722			
Lin	1	8.191	41.889	**0.000**			
Pupil-size Stimulus	1	116.778	1.668	****0.199			
Own/Other Species	1	116.898	0.948	0.332			
Quad	1	21.019	32.423	**0.000**			
Quad * Pupil-size Stimulus	1	108.521	4.790	**0.031**			
Quad * Own/Other Species	1	100.250	0.438	0.510			
Quad * Pupil-size Stimulus * Own/Other Species	1	101.117	4.631	**0.034**			
Cub	1	6.096	11.280	**0.015**			

Estimates of Covariance Parameters							
Parameter						95% Conf. Interv.	
		Estimate	Std. Error< Error/p>	Wald Z	Sig.	Lower Bound	Upper Bound
Repeated Measures	AR1diag gdiag.	0.040	0.002	2.374	**0.000**	0.040	0.002
	AR1rho	0.981	0.001	1038.617	**0.000**	0.981	0.001
Int+ Lin+ Quad+Cub	Int	0.021	0.004	5.653	**0.000**	0.021	0.004
[subject = id *Stimulus Nr]	Lin	0.330	0.145	2.265	**0.024**	0.330	0.145
	Quad	0.334	0.093	3.614	**0.000**	0.334	0.093
	Cub	0.241	0.059	4.068	**0.000**	0.241	0.059
Int+ Lin+ Quad+Cub	Int	0.009	0.005	1.672	0.095	0.009	0.005
[subject = id]	Lin	0.424	0.248	1.711	0.087	0.424	0.248
	Quad	0.181	0.113	1.604	0.109	0.053	0.615
	Cub	0.074	0.060	1.251	0.211	0.016	0.359

### Eye Movements

It is possible that human observers viewed the faces differently than the chimpanzees and that that caused the difference in the pattern of the pupillary response. To rule out this explanation, we analyzed the relative looking time on the eyes, by dividing the absolute looking time on the eye-region by the absolute looking time on the whole computer screen. The statistical model was the same as the pupil-mimicry model described above, but without the three polynomials. We observed a main effect of Species Subject showing that humans attended longer to the eye region of the observed stimuli than chimpanzees (*p* = .007). Importantly, this was independent of Stimulus Pupil-size or Own/Other Species Stimulus.

Including looking times in the analysis of pupil-size yielded very similar results to the results discussed above. In short, there was no confounding of eye movements on species differences in pupil-mimicry.

## Discussion

The findings of the current study demonstrate that both humans and chimpanzees mimic the pupil-size, especially when viewing their own species, suggesting that the sharing of focused attention at close range and the recognition and mimicry of subtle and autonomic signals such as pupil dilation enhances affiliative behavior and social bonding between conspecifics. It has been suggested that the appearance of eye-white was the starting point of linkage between two individuals on the level of eye-signals [33]. However, the current findings show that pupil-mimicry is not uniquely human but *is possible* in a species that has no clear visible eye-white and has less communicative eyes than humans. Nonetheless, eye-white most probably still *helped* observers in detecting salient and biologically relevant cues from the eyes and evolved for that exact purpose [50]. This viewpoint supports the somewhat stronger effects of mimicry in the human as compared to the chimpanzee subjects in our study.

The within-species effect on pupil-mimicry that was observed in the current study suggests that the function of pupil-mimicry may be similar to other forms of mimicry. Mimicry in general grounds positive feelings and impacts on the bond between individuals [9]. Even linkage on the physiological level with a close partner predicts satisfaction about the relationship [13]. Mimicry facilitates cooperation and occurs in mammals and birds, species with complex social networks that care for their young [51]. Previous studies have shown that *social relevance* is crucial for mimicry to occur [8]. In humans, it has been shown that attractiveness, friendship, and social status can enhance mimicry while social stigma, negative mood, and out-group membership can inhibit mimicry [52]. Social relevance can be directly modulated by eye contact. For example, action mimicry decreases when the gaze of the observed is averted [53], [54]. The *relevance* of an eye-signal is also dependent on *whom* is signaling it, whether it is someone familiar or unfamiliar and as we show here, whether the observed belongs to one's own species as compared to another species. From evolutionary perspective, it is more adaptive to establish and maintain social bonds with members of your own species than with members of a different species because there is a higher probability of (future) contact with in-group members. Therefore, mimicry with one's own species is more relevant than mimicry with members of another species. Relevance may also differ between individuals within a group. We here observed that all three chimpanzee mothers synchronized specifically with chimpanzees. For the survival of their young, chimpanzee mothers (and human mothers alike although we did not have them in our sample) strive after stable relationships and seek support and protection from group members, possibly especially with synchronizing partners. It could be that especially the chimpanzee mothers, by great experience learned to make use of pupil-synchronization. How, in terms of neural mechanisms, pupil-synchronization is regulated and how factors such as experience may impact upon it is uncertain but the amygdala is likely to play an important role.

The amygdala responds strongly to socially relevant cues of conspecifics. This area signals when being confronted with an out-group member [55], processes emotions [56] and detects changes in anothers pupil size [57]. The ability to quickly respond to such salient cues has been attributed to an evolutionary old subcortical route for processing of emotional information [59], [60]. Previous research with blind-sight patients has shown that the mimicry of facial expressions is possible via this subcortical route. Patients that could not consciously perceive presented emotional expressions, did unconsciously and very rapidly synchronize their facial expression [61]. In the current study, subjects' pupil-size very rapidly adapted to the observed pupil-size. This new finding, together with previous findings suggesting that the amygdala might regulate pupil-mimicry [18], [19], suggests the involvement of a sub-cortical pathway [60]. Two possible routes to pupil-mimicry might be via the superior culliculi, the pulvinar, and the amygdala, which projects to different brain stem areas that induce pupil mimicry [20], [58], or from the superior colliculus directly to the brainstem nuclei that subsequently project to the amygdala [62]. With the low temporal resolution of fMRI, it is difficult to get direct insight into this ancient route although it might solve part of the puzzle. Studying pupil-mimicry in blind-sight patients could provide further understanding in the neural underpinnings and more specifically about the role of the subcortical route.

Our study has several limitations. First of all, in real life, ambient light changes constantly and therefore the real-life impact of pupil-mimicry remains unknown. That brings us to the second limitation, which is the ecological validity of the set-up of this study. It is possible that chimpanzees show stronger pupil-mimicry during real-world social interactions with familiar others that have direct consequences for them. Studying two individuals at the same time during an interaction is an interesting future avenue [63]. We found not only stronger pupil-mimicry effects in the human sample, but also a greater difference between mimicking ones own vs. the other species in human as compared to chimpanzee subjects. It is possible that stronger effects would have been found in the chimpanzees had they been less familiarized to humans or had they been brought into a competitive setting. Moreover, whether within vs. cross-species effects translate to in-group/out-group differentiation as described in for example the facial mimicry literature, is uncertain. Future studies in humans with different in-group/out-group manipulations can give more insight into this question.

Evolutionary theory implies that the propensity to mimic pupil-size should be especially adaptive within groups. In line with this assumption, pupil-mimicry is shared among humans and chimpanzees and is stronger during interactions with members of one's own species than during interactions with members of the other species. Humans most likely evolved their communicative eyes with clear eye-white and fine musculature precisely because it benefits within-group interactions, survival, and prosperity.
